# Optimal Dosage of Platelet-Rich Plasma Injections in Patients With Osteoarthritis of the Knee: A Scoping Review

**DOI:** 10.7759/cureus.75497

**Published:** 2024-12-10

**Authors:** Ambika Singh, Sarthak Chakravarty, Dylan Sehgal, Brandon Rust, Khavir A Sharieff

**Affiliations:** 1 Osteopathic Medicine, Nova Southeastern University Dr. Kiran C. Patel College of Osteopathic Medicine, Fort Lauderdale, USA; 2 Surgery, Nova Southeastern University Dr. Kiran C. Patel College of Osteopathic Medicine, Tampa, USA

**Keywords:** dosage, intra-articular injection, knee, osteoarthritis, platelet-rich plasma

## Abstract

Knee osteoarthritis (KOA) is a healthcare burden affecting over 595 million people worldwide. Recently, intra-articular platelet-rich plasma (PRP) injections from the patient's blood have shown promise in slowing KOA progression due to platelets’ regenerative properties. This study aimed to evaluate the optimal dosing and schedule for PRP therapy in managing mild to moderate KOA. A systematic search was conducted across Embase, Ovid Medline, Web of Science, Cochrane Central, and CINAHL using the Preferred Reporting Items for Systematic Reviews and Meta-Analyses (PRISMA) guidelines to identify articles published from August 2015 to March 2024. Keywords included “platelet rich plasma,” “knee osteoarthritis,” and “administration schedule.” Inclusion criteria were studies on human patients utilizing PRP as monotherapy in experimental trials, while review articles, editorials, case reports, and meta-analyses were excluded. Three reviewers independently extracted and described patient interventions and outcomes, focusing on Western Ontario and McMaster Universities Osteoarthritis Index (WOMAC), Visual Analog Scale (VAS), and imaging changes. Thirty-nine publications with PRP monotherapy protocols were found, with fourteen meeting the inclusion criteria. Twelve studies were randomized clinical trials, and two were longitudinal cohort studies, totaling 1704 patients with a mean follow-up of 7.51 ± 4.82 months. The most common PRP protocol was 4.357 ± 1.419 mL infusions, with three doses every four weeks and a single dose being frequent. Platelet values varied, with seven including a mean platelet count, three reporting that the platelet concentration in each dose had to be at least 150,000/μL, and four did not include platelet concentration. There was notable variation in PRP acquisition protocols, blood volume, and centrifugation processes across studies. Therapeutic benefits were represented by WOMAC and VAS scores rather than imaging changes. PRP injections appear to be safe and effective for symptomatic relief of knee pain associated with mild to moderate osteoarthritis (OA). The average infusion volume was 4 mL, administered at three doses four weeks apart. Given that platelet-derived growth factors promote the proliferation of chondrocytes and mesenchymal stem cells, leading to the stimulation of articular cartilage remodeling, further studies are warranted to assess the optimal platelet count necessary for the long-term effects of PRP in knee cartilage healing and sustained symptomatic improvement.

## Introduction and background

Osteoarthritis (OA) is a progressive and debilitating degenerative articular cartilage disorder that affects millions worldwide. Knee osteoarthritis (KOA), in particular, is a multifactorial condition influenced by both age and genetic predisposition, involving changes in the underlying bone structure and surrounding soft tissue [1,2]. Affecting over 20% of the population above the age of forty, KOA represents a significant burden on healthcare systems globally [1,2]. Current treatments, including analgesics, non-steroidal anti-inflammatory drugs (NSAIDs), physical therapy, and arthroplasty, primarily aim to relieve symptoms and improve joint function [3].

Recently, there has been a surge of research into intra-articular injections within the field of orthopedic regenerative medicine, exploring potential solutions for long-term symptom relief and enhanced joint functionality [3]. Among these emerging regenerative techniques, platelet-rich plasma (PRP) therapy has gained considerable attention [4]. PRP involves the autologous transplantation of platelet-concentrated blood products. Numerous studies have demonstrated the clinical effectiveness of PRP therapy, particularly in KOA patients. By centrifuging the blood, a concentration of bioactive platelets is obtained, resulting in a mix of growth factors and cytokines associated with reduced tissue inflammation [5,6]. These components are then injected into the affected joint to leverage the regenerative capacity of platelets and stimulate repair mechanisms within the osteoarthritic joint.

Despite the growing interest in PRP therapy for KOA, comprehensive investigations of its clinical efficacy, safety profile, and optimal dosing remain crucial for establishing its effectiveness. Current studies have compared the effectiveness of PRP with that of other injection types, such as hyaluronic acid and corticosteroid injections [1,7]. However, the results have been inconsistent, with some studies demonstrating significant improvements in pain and function with PRP, while others show no significant differences compared to placebo or other treatments [8].

Several factors may contribute to this variability. First, dosing and injection protocols vary widely across studies, including differences in the number of injections, time intervals between injections, and PRP preparation methods [9]. For example, some studies use a single injection [1,10-12], while others administer multiple injections over varying time frames [5,6,13-20]. Secondly, patient characteristics such as age, severity of KOA, and body mass index (BMI) can influence the treatment outcomes [21]. Additionally, the follow-up duration across studies ranges from a few months to over a year, potentially impacting observations of PRP’s long-term efficacy. The majority of the articles had a 12-month follow-up [1,6,10,13,17-20], three articles had a six-month follow-up [11,12,14], and the rest of the four articles had a follow-up of either 60 months [5], 24 months [15], three months [16], or 12 weeks [20].

Notably, several studies report non-significant effects of PRP compared to normal saline injections [8]. The contradicting findings highlight the need for further research to understand PRP’s mechanisms of action and identify the patient populations most likely to benefit from PRP therapy [22]. The multifactorial nature of KOA underscores the need for effective interventions to alleviate the disease burden on the population.

Despite numerous studies, a significant gap persists in standardized dosing and protocols. Existing research offers varying perspectives on the optimal dosing schedule and dosage, highlighting the need for consensus in the field. Therefore, this study aims to identify the optimal dosing and schedule of PRP therapy in KOA management through a scoping review of the current literature.

## Review

Methods

Study Design and Search Strategy

On March 2024, a search was executed across Embase, Ovid Medline, Web of Science, Cochrane Central, and CINAHL employing the Preferred Reporting Items for Systematic Reviews and Meta-Analyses (PRISMA) guidelines of 2020. The PRISMA guidelines were utilized to ensure a structured and rigorous approach to evidence synthesis for this scoping review [23]. The search string was as follows: (“Plama, Platelet-Rich” OR “Platelet Rich Plasma”) AND (“Knee Osteoarthritides” OR “Knee Osteoarthritis” OR “Osteoarthritis of Knee” OR “Osteoarthritis of the Knee”) AND (“Dos*” OR “volume” OR “dose response”) AND (“Administration Schedule, Drug” OR “Administration Schedules, Drug” OR “Drug Administration Schedules” OR “Schedule, Drug Administration” OR “Schedules, Drug Administration”).

Following the Joanna Briggs Institute (JBI) methodology for scoping reviews, we applied the Population, Concept, and Context (PCC) framework to define our search parameters [23]. The target population was human patients with KOA. The concept focused on the optimal dosage and administration intervals of PRP, with the context being experimental trials published in English before March 2024.

Study Criteria

All studies investigating the optimal dosage and administration intervals of PRP in human patients with KOA, with a publication year cut-off of March 2024, were included in the review. Eligible studies had to meet the following criteria: target the human population, utilize PRP as a monotherapy, be conducted as experimental trials, and be written in English. Review articles, editorials, case reports, and meta-analyses were excluded from the review. 

**Figure 1:**
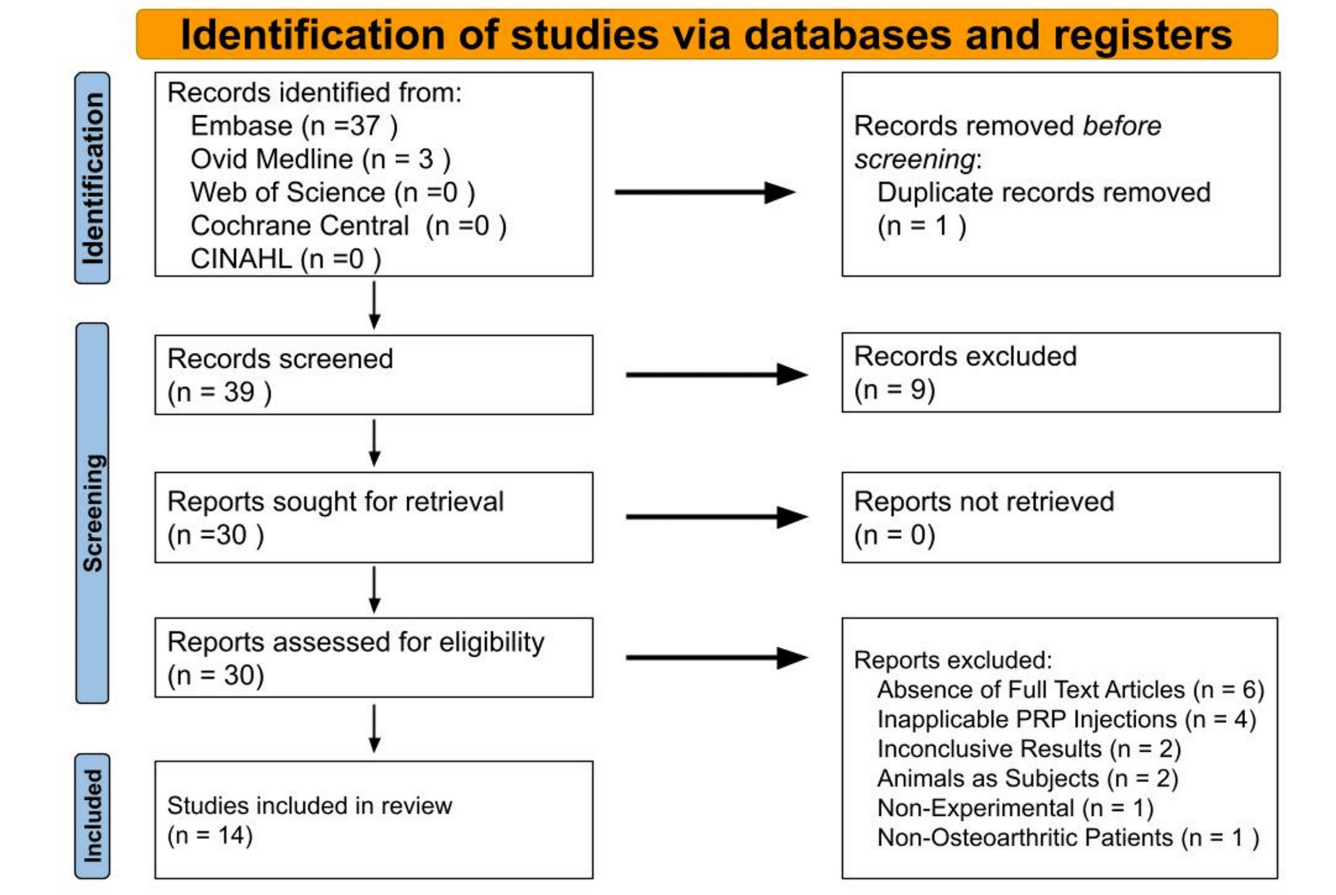
Preferred Reporting Items for Systematic Reviews and Meta-Analyses (PRISMA) flowchart.

References retrieved from the databases were managed using Rayyan (Rayyan Systems Inc., California, MA) an online software designed for screening large volumes of literature for systematic and scoping reviews [24]. Three independent reviewers initially performed a blinded review of all references in a two-tiered screening process using Rayyan. Articles that met exclusion criteria were discarded. Abstracts meeting the inclusion criteria were further reviewed in full text by the same reviewers, maintaining a blinded approach throughout the process. The study selection process, in accordance with PRISMA guidelines, is illustrated in Figure 1.

Search Results

After conducting the literature search, 39 articles were initially identified and screened for appropriateness. Nine articles were excluded because they targeted the wrong population, did not utilize PRP, or combined PRP with other substances for the injection. Of the remaining 30 articles, 16 were excluded because they either used animal subjects or the full-text articles were not accessible online. Ultimately, 14 studies met our inclusion criteria and were included in the review.

Of the 14 included studies, 12 (85.7%) were randomized controlled trials, and 2 (14.3%) were longitudinal studies. Among these, 8 (57.1%) were single-blinded randomized clinical trials (RCTs) and 4 (28.6%) were double-blinded. The Western Ontario and McMaster Universities Osteoarthritis Index (WOMAC) score and visual analog scale (VAS) were utilized in 11 (78.6%) studies. The WOMAC score is a subjective measure that evaluates patients' symptoms and functionality, including sections for pain, stiffness, and physical function, each with its own subsections. The VAS score allows patients to indicate their pain severity on a linear scale, with 10 cm representing "worst pain" and 0 cm representing "no pain." Additional outcome measures included the International Knee Documentation Committee (IKDC), Knee Injury and Osteoarthritis Outcome Score (KOOS), Tegner and Marx scoring systems, Lequesne scale, Lysholm and Tegner scale, and American Knee Society Score (AKSS) (see Table 1). Demographic data of the patients from the included studies were detailed in Table 2.

**Table 1:** Breakdown of the fourteen included articles. PRP: platelet-rich plasma, WOMAC: Western Ontario and McMaster Universities Osteoarthritis Index, VAS: visual analog scale, IKDC: International Knee Documentation Committee, AKSS: American Knee Society Score, RPM: revolutions per minute, PROMs: patient-reported outcome measures.

Author	Centrifuge protocol	Dosage quantity	Platelet count	mL’s of PRP injected	Follow-up period	PROMS
Gobbi et al., 2015 [15]	3500 RPM, 5 mins	First cycle: three injections, four weeks apart; second cycle: same as the first cycle administered after one year	Not described	4 mL	24 months	KOOS, VAS, Tegner and Marx
Smith2016 [19]	5 mins, RPM not described	Three doses, one week apart	Not described	3-8 mL	12 months	WOMAC
Duymus et al., 2017 [13]	3700 RPM, 7 mins	Two doses, four weeks apart	1.5 x 10^6^/uL	5 mL	12 months	WOMAC and VAS
Lisi et al., 2018 [17]	900 RPM, 7 mins	Three doses, four weeks apart	Not described	Not described	12 months	WOMAC, Lysholm, Tegner, AKSS, Lequesne, VAS
Buendía-López et al., 2018 [1]	First spin: 1050 RPM, 15 mins; second spin: 2000 RPM, 10 mins	Single dose	1.095 x 10^6^/mm^3^	5 mL	12 months	WOMAC and VAS
Sucuoğlu et al., 2019 [20]	Not described	Three doses, three weeks apart	Not described	Not described	12 weeks	VAS
Ayeni et al., 2019 [14]	First spin: 1800 RPM, 15 mins; second spin: 2500 RPM, 15 mins	Three doses, four weeks apart	Not described	3 mL	Six months	WOMAC and VAS
Raeissadat et al., 2020 [18]	Not described	Two doses, three weeks apart	Not described	Not described	12 months	VAS, WOMAC, Lequesne
Lacko et al., 2021 [16]	First spin: 3200 RPM, 15 mins; second spin: 1500 RPM, 10 mins; third spin: 3200 RPM, 10 mins	Three doses, one week apart	Not described	3 mL	Three months	WOMAC and VAS
Bansal et al., 2021 [10]	Not described	Single dose	Mean: 14.38 ± 1.76 × 10^5^ platelet/µl	8 mL	12 months	WOMAC
Subramanyam et al., 2021 [6]	3500 RPM, 5 mins	Single, double, and triple dose groups. Injected two weeks apart for repeat injection groups.	Not described	4 mL	12 months	VAS
Chu et al., 2022 [5]	First spin: 3200 RPM, 5 mins; second spin: 3300 RPM, 3 mins	Three doses, one week apart	832.1 ± 269.3 × 10^9^/L	5 mL	60 months	WOMAC, VAS, and IKDC
Wang et al., 2022 [12]	3200 RPM, 6 mins	Single dose	Not described	4 mL	Six months	WOMAC
Kothari et al., 2023 [11]	First spin: 1500 RPM, 15 mins; second spin 3000 RPM, 10 mins	Single dose	Not described	2-3 mL	Six months	VAS, WOMAC, IKDC

**Table 2:** Demographic data of the patients from the included studies. *Kellgren-Lawrence; **Shahriaree Classification System. KOA: knee osteoarthritis, WOMAC: Western Ontario and McMaster Universities Osteoarthritis Index, NSAID: non-steroidal anti-inflammatory drug, OA: osteoarthritis.

Author	Total number of patients	Female:Male ratio	Stage of OA	Patient population age (years)	BMI (kg/m^2^)	Other inclusion criteria
Gobbi et al., 2015 [15]	93	39:54	Grade 1-2*	Range: 40-65	<30	Patients having severe pain without relief with anti-inflammatory agents after three months; stable knees without malalignment or maltracking of the patella; severe pain without relief with anti-inflammatory agents even after three months; platelets between 150,000 and 450,000/μL; no surgery on target knee within two years prior to first injection; zero, trace or 1+ effusion on stroke test grading scale
Smith2016 [19]	30	19:11	Grades 2-3*	Range: 30-80	28.50 ± 5.91	Documented diagnosis of primary OA for at least six weeks; continued OA pain in target knee despite at least six weeks of nonoperative treatments (activity modification, weight loss, physical therapy, NSAID); WOMAC score of at least 8/20 and at least moderate pain for at least two questions on WOMAC function subscale
Duymus et al., 2017 [13]	102	96:6	Grade 2-3*	Range: 47-80	<30	Stable knees without malalignment; normal blood results and coagulation profile
Lisi et al., 2018 [17]	58	22:36	Level 2**	>18	Not described	No previous OA treatment with local hyaluronic acid or steroid injections; life expectancy >1 year; no ongoing pregnancy; ability to understand and complete clinical functional scales; no diagnosed allergy to hyaluronic acid; no acute bacterial skin and soft structure infection of the knee
Buendía-López et al. 2018 [1]	98	51:47	Grade 1-2*	Range: 50-63	23.8-26.1	Symptomatic KOA defined by Spanish Society of Rheumatology
Sucuoğlu et al., 2019 [20]	42	37:5	Grade 2-4*	Range: 40-80	28.5 ± 9.71	Pain for more than three months
Ayeni et al., 2019 [14]	45	16:29	Grade 1-2*	Range: 41-85	28.1 ± 6.0	N/A
Raeissadat et al., 2020 [18]	200	139:61	Grade 2-3*	Range: 50-75	28.24 ± 2.8	Knee pain and symptoms for longer than three months
Lacko et al., 2021 [16]	36	22:14	Grade 1-3*	Mean: 53.4 ±7.7	29.1 ± 3.4	Chronic moderate to severe pain in the affected knee
Bansal et al., 2021 [10]	132	51:81	Grade 1-3*	≥ 50	Not described	Symptomatic KOA with pain for at least three months; less than 30 min of morning stiffness; analgesics usage at least once a week
Subramanyam et al., 2021 [6]	90	68:22	Grade 1-2*	Range: 36-60	Not described	Bilateral KOA
Chu et al., 2022 [5]	610	360:250	Grade 1-3*	Range: 18-80	<40	Knee pain on most days in the last month; unilateral symptoms; damage to articular cartilage seen on weight-bearing radiographs or MRI
Wang et al., 2022 [12]	110	82:28	Grade 1-3*	>50	24.04 ± 2.90	Diagnosis of primary KOA
Kothari et al., 2023 [11]	40	26:14	Grade 1-2*	>50	Not described	Symptomatic KOA; little pain relief or no pain relief after <2 weeks of conservative treatment; normal platelet count

Results

Platelet-Rich Plasma (PRP) Acquisition Protocol and Dosage

From the 14 studies, 1704 patients were analyzed using PRP as a test preparation. The quantity of PRP administered to the affected knee ranged from 2 to 8 mL, with an average of 4.357 ± 1.419 mL. A significant point of contention among the included studies is the acquisition protocol for PRP. While all studies agreed on obtaining blood from a venous blood draw, the volume of blood drawn varied from 8 mL to 50 mL [5,6,15]. Additionally, there is no standard centrifugation process for obtaining the highest yield of PRP. Among the studies that fully described their protocols, five (35.7%) performed a single centrifugation step [6,12,13,15,17], four (28.6%) performed two centrifugations [1,5,11,14], and one (7.1%) performed triple centrifugation [16]. Even within groups using the same number of centrifugations, there was no consensus on the revolutions per minute (RPM) or duration needed to prepare the PRP, with centrifugation times ranging from three to 15 minutes. All studies utilizing multiple centrifuge protocols agreed on a final spin set at a higher RPM than the initial spin.

The administration of PRP ranged from a single dose to three doses. Specifically, four (28.6%) studies administered PRP as a single dose [1,10-12]. Three (21.4%) studies administered three doses at four-week intervals [14,15,17], and three (21.4%) studies administered three doses at weekly intervals [5,16,19]. One (7.1%) study administered two doses at three-week intervals [18], one (7.1%) study administered three doses at three-week intervals [20], and one (7.1%) study administered two doses at four-week intervals [13]. Finally, one (7.1%) study had three experimental groups consisting of single, double, and triple doses administered at two-week intervals [6]. The minimum follow-up period after treatment was 15 days, and the maximum was 60 months, with a mean follow-up of 7.51 ± 4.82 months.

Out of the 14 studies, seven (50%) included a mean platelet count [1,5,6,10-13], four (28.6%) did not include platelet concentration [14,16,18,19], and three (21.4%) specified that the platelet concentration in each dose had to be at least 150,000/μL [12,15,20]. Specifically, five (35.7%) studies reported platelet values as a mean platelet count ranging from one million to 832 billion [1,5,6,10,13], five (35.7%) reported platelet concentrations ranging from 150,000 to 450,000/μL [11,12,15,17,20], and four (28.6%) did not report any platelet values [14,16,18,19].

All included studies indicated improvement in secondary scoring systems in favor of PRP compared to control treatments. Every study concluded that PRP injections resulted in short-term and long-term clinical improvement in patients [1,5,6,10-20].

Radiological Findings

Five (35.7%) of the 14 studies evaluated radiological changes in patients at baseline and after treatment [1,5,10,17,19]. Plain radiographs were used to determine Kellgren-Lawrence (KL) grades, and magnetic resonance imaging (MRI) was used to assess changes in the mean cartilage volume in the knee joint. Only one study reported an improvement in cartilage volume in patients who received PRP [17]. In this study, 14 out of 30 patients (48.3%) showed greater than one-grade improvement in cartilage volume as determined by MRI. The other four studies did not find improvements in knee cartilage volume or KL grades. These four studies noted that the progression of OA continued in both the treatment and control groups [1,5,10,17]. However, they also highlighted that the disease progression was worse in the non-PRP groups compared to the PRP groups, indicating that PRP treatment was associated with slower disease progression.

Biomarkers

Of the 14 studies included, three (21.4%) evaluated levels of pro-inflammatory and anti-inflammatory biomarkers [5,10,16]. All three studies found that PRP had a net anti-inflammatory effect. Lacko et al. studied the levels of 19 biomarkers with varying properties. They found an increase in specific pro-anabolic and anti-inflammatory biomarkers, alongside a concomitant decrease in pro-inflammatory biomarkers, three months after three intra-articular applications of PRP [16]. Chu et al. found that the levels of IL-1β and TNF-α in synovial fluid were lower in the PRP group at six months post-treatment but returned to baseline at 12 months [5]. Bansal et al. observed a significant decline in IL-6 and TNF-α levels from baseline in the PRP group (P < 0.05) compared to the hyaluronic acid (HA) group at one month [10]. However, no significant differences were observed in IL-8 levels between the treatment and control groups.

Discussion

KOA is a degenerative articular cartilage joint disorder with significant consequences for the healthcare system. Current non-operative treatments, such as analgesics, orthotics, and physical exercise, may offer only slight symptom relief, prompting a surge in research into regenerative medicine techniques, such as PRP therapy [1,2,5]. PRP involves autologous blood concentrations of cytokines and growth factors that are believed to reduce inflammation and stimulate tissue regeneration [4-6]. Addressing the gap in dosing and scheduling in PRP research is critical for improving OA management and reducing its burden on society. This study sought to bridge these gaps by conducting a scoping review of the current literature.

The 14 included studies showed wide variations in PRP acquisition and preparation methods. Of those describing their protocols, five studies used a single centrifugation protocol [6,12,13,15,17], whereas the other five studies used multiple centrifugation protocols [1,5,11,14,16]. The centrifugation durations varied from a single spin for seven minutes to triple centrifugation for a cumulative 35 minutes, with speeds ranging from 1050 RPM to 3300 RPM. Obtaining efficacious PRP injections depends significantly on the preparatory process, as platelets are fragile cells that can be altered by vigorous centrifugation [25].

Regarding injection frequency, there were substantial differences in the number of injections and intervals between them. Four studies administered a single dose [1,10-12]. Among the 10 studies that administered multiple doses, three administered three doses at four-week intervals [14,15,17], three administered three doses at weekly intervals [5,16,19], one administered two doses at three-week intervals [18], one administered three doses at three-week intervals [20], and one administered two doses at four-week intervals [13]. The last study consisted of three experimental groups consisting of a single, double, and triple dose, in which the multiple injection groups received their injections at two-week intervals [6]. A study comparing the clinical effectiveness of single versus multiple PRP injections found that a single injection was as effective as multiple injections within a six-month interval for pain improvement, as measured using the VAS or visual numeric scale (VNS) [26]. However, multiple injections were more effective for functionality improvement, as measured by the WOMAC or IKDC scale. Thus, a multi-dose regimen may offer symptomatic relief, making PRP a safer and more practical approach than other cell-based therapies [27].

All studies found that PRP conferred symptomatic benefits to patients, predominantly based on the WOMAC index and VAS scores. Secondary scoring methods, such as the IKDC and KOOS scales, showed similar trends. When considering objective measures such as imaging, five out of the 14 studies measured imaging changes [1,5,10,17,19]. Only one study found a significant improvement in the mean cartilage volume on MRI [17], while the other four studies noted worsening OA in all patients, with disease progression being worse in control groups compared to experimental groups. An RCT assessing MRI changes after PRP injections in KOA found that patellofemoral cartilage volume and synovitis were better in the PRP group [28]. This emphasizes the need for studies to include objective measures such as imaging to understand the anatomical changes associated with PRP. Since few studies have used imaging modalities, it is challenging to confirm decisively that PRP injections anatomically affect KOA.

In the three studies measuring inflammatory biomarkers, PRP injections increased the levels of anti-inflammatory biomarkers and/or reduced levels of pro-inflammatory biomarkers [5,10,16]. For example, the concentrations of IL-17A, IL-1β, TNF-α, RANKL, IL-6, and IFN-γ were significantly downregulated in the PRP treatment group compared to those in the placebo group after an eight-week observation [29]. More studies with this form of affirmation are crucial to promoting the effectiveness of PRP injections. Understanding the anatomical and physiological changes associated with PRP is essential for creating a standardized PRP dosage for the general population.

PRP injection is a fundamentally safe therapy due to its autologous nature, which reduces concerns over transmissible diseases such as human immunodeficiency virus (HIV), Hepatitis B and C [27]. However, each injection carries risks, including reactions at the injection site, infection, or possible allergic reactions. Proper precautions of sterility, such as the use of sterile gloves, antiseptic solutions, and masks, are necessary to avoid infection. Current studies highlight PRP's inability to support bacterial growth because of its slightly acidic nature [28,30-32]. Although uncommon, patients can develop allergic reactions to components of PRP preparation. Without standardized protocols, careful consideration must be taken when administering PRP therapy.

This scoping review highlights potential limitations in data available for reporting on the optimal dosage of PRP for efficacy assessment. Additionally, there were inconsistencies in the reporting of the blood composition and factors across studies. Some studies provided specific platelet counts for patients, whereas others reported mean platelet counts for the entire study population, complicating the standardization of PRP composition. The studies also varied in dosages and administration intervals, with some using single doses and others using multiple doses at different intervals. Despite the overall positive patient outcomes with PRP injections, these divergent approaches hinder the identification of an optimal PRP composition and administration schedule for maximal efficacy.

As PRP injections gain popularity, understanding the healing process is of paramount importance. However, variations in administration and blood composition hinder the standardization of results across patient populations. Therefore, studies should quantify platelet counts in each PRP injection, whether single- or multi-dose. Comparing pre- and post-injection inflammatory factors can reveal correlations with the injection process. Additionally, imaging pre- and post-injections provides insights into anatomical changes. Although subjective patient reports are valuable, they alone are insufficient for establishing a universal PRP dosage.

The review was limited by the small number of studies with reportable data on effective dosing and variability in reported blood composition factors. Despite these limitations, our data suggests that PRP injections can offer beneficial relief to patients. However, determining how to extend this relief to a broader patient population still remains a challenge.

The findings of this review highlight the need for future research and practice guidelines for physicians using PRP in patients with KOA to optimize outcomes. Additionally, future studies should focus on standardizing PRP preparation and administration protocols to maximize their applicability and efficacy.

## Conclusions

This scoping review investigated 14 studies on the optimal dosing and dose schedule of PRP injections in patients with mild-to-moderate KOA. Our review indicates that PRP treatment provides symptomatic relief as measured by subjective tools like the WOMAC index and VAS scores. Additionally, MRI analysis suggests that PRP injections may help diminish the progression of disease severity. However, variations in dosing, preparation, and administration across the studies highlight the need for further research to establish standardized protocols for PRP usage.
